# Neuromedins NMU and NMS: An Updated Overview of Their Functions

**DOI:** 10.3389/fendo.2021.713961

**Published:** 2021-07-01

**Authors:** Ludwik K. Malendowicz, Marcin Rucinski

**Affiliations:** Department of Histology and Embryology, Poznan University of Medical Sciences, Poznan, Poland

**Keywords:** neuromedin U (NMU), neuromedin S (NMS), GPCR (G protein coupled receptor), biologically active peptides, NURP, NSRP, NMUR1, NMUR2

## Abstract

More than 35 years have passed since the identification of neuromedin U (NMU). Dozens of publications have been devoted to its physiological role in the organism, which have provided insight into its occurrence in the body, its synthesis and mechanism of action at the cellular level. Two G protein-coupled receptors (GPCRs) have been identified, with NMUR1 distributed mainly peripherally and NMUR2 predominantly centrally. Recognition of the role of NMU in the control of energy homeostasis of the body has greatly increased interest in this neuromedin. In 2005 a second, structurally related peptide, neuromedin S (NMS) was identified. The expression of NMS is more restricted, it is predominantly found in the central nervous system. In recent years, further peptides related to NMU and NMS have been identified. These are neuromedin U precursor related peptide (NURP) and neuromedin S precursor related peptide (NSRP), which also exert biological effects without acting *via* NMUR1, or NMUR2. This observation suggests the presence of another, as yet unrecognized receptor. Another unresolved issue within the NMU/NMS system is the differences in the effects of various NMU isoforms on diverse cell lines. It seems that development of highly specific NMUR1 and NMUR2 receptor antagonists would allow for a more detailed understanding of the mechanisms of action of NMU/NMS and related peptides in the body. They could form the basis for attempts to use such compounds in the treatment of disorders, for example, metabolic disorders, circadian rhythm, stress, etc.

## Introduction

It has been 35 years since the isolation from pig spinal cord by Minamino et al. of numerous small neuropeptides whose common feature was the stimulation of smooth muscle contraction (1). These were named neuromedins, and neuromedin U (NMU) is one of them. NMU exerts a potent contractile effect on the muscles of the rat uterus, hence the derivation of its name (U - uterus).

In the 1990s, the structure of NMUs in various species was recognized and their synthesis was elucidated. NMU is a highly conserved neuropeptide present in many species, existing as multiple isoforms. Moreover, specific binding of the neuropeptide to various organs was demonstrated. The presence of NMU-like immunoreactivity was also reported (RIA and immunohistochemistry) in a number of human organs and various animal species. Major physiological effects in which NMU is involved have also been identified. These include smooth muscle contraction, increased blood pressure, gastric emptying and modification of intestinal ion transport and motility (2).

With the introduction of modern molecular biology techniques, at the turn of the 20th and 21st century, specific NMU receptors (NMUR1 and NMUR2) were identified and characterized. Their distribution in the body and intracellular signaling pathways regulated by these receptors has been recognized. In parallel, NMU was shown to be involved in the regulation of body metabolism, exhibiting anorexigenic effects. This last observation has greatly increased the interest of numerous research centers in the physiological role of this neuromedin.

Another significant discovery related to NMU was the isolation of neuromedin S (NMS) from rat brain. Both NMU and NMS are endogenous ligands for NMUR1 and NMUR2 receptors (3, 4). Since then, many studies have focused on comparing the physiological effects of both neuropeptides in different cells and organisms. The precursors of NMU and NMS (preproNMU - ppNMU and preproNMS - ppNMS) share high structural similarity, and as shown by Mori et al. (5) and Ensho et al. (6), other peptides may also originate from them, which they named “neuromedin U precursor related peptide” (NURP) and “neuromedin S precursor related peptide” (NSRP) (7). In this context, it should be noted that already in 2009 Bechtold et al. showed that mice proNMU104-136, actually named NURP33, is involved in the regulation of energy homeostasis (8). Currently, several groups are undertaking research on the role of these peptides in the regulation of various biological processes.

Several review papers have been published in the literature on NMU, NMS and their receptors as well as their physiological role. In this context, the contributions by Brighton et al. (2), Budhiraja and Chugh (9), Mori et al. (4), Mitchell et al. (10), Malendowicz et al. (11), Martinez and O’Driscoll (12), and Alhosaini et al. (13) deserve to be mentioned. On the other hand, the aim of the current review is to provide readers with the recent data on the physiological role of NMU and NMS action, with a particular focus on the differences in the action of these neuromedins, at both cellular and organ levels. In this review, our attention will focus mainly on the human, rat and mouse data.

## Synthesis of preproNMU and preproNMS and Structure of Related Peptides

### Genes Encoding ppNMU and ppNMS and Their Transcripts

**Figure 1:**
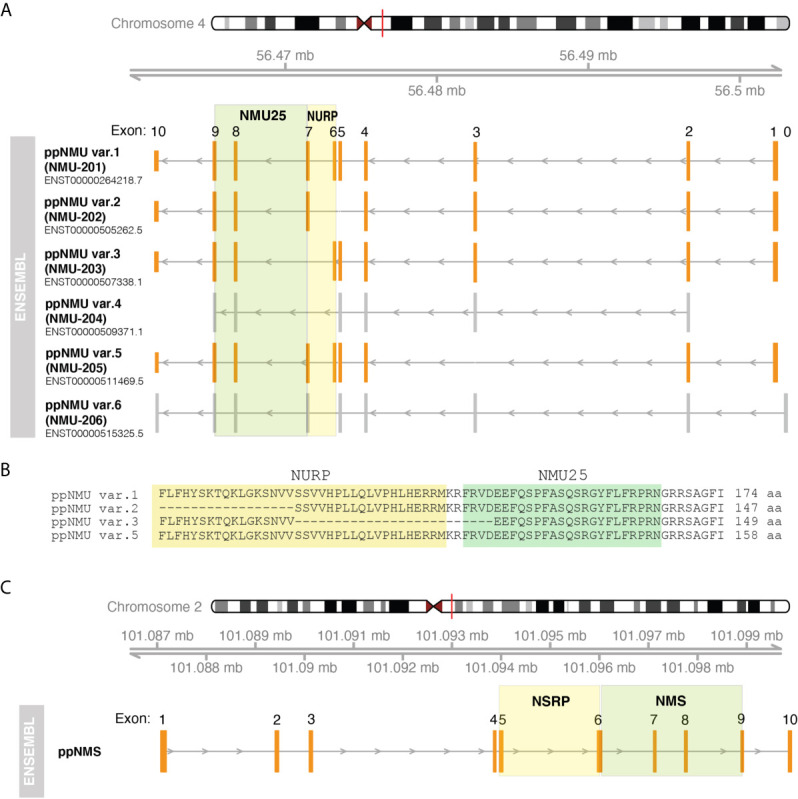
Structure of human genes encoding *ppNMU* **(A)** and *ppNMS* **(C)** according to ENSEMBL database. The structure of individual genes or alternative splice variants and their positions on the chromosomes was visualized using “GenomicFeatures” and “Gviz” packages of the programming R language. Exons of protein-coding splice variants are marked in orange whilst grey color corresponds to exons of non-protein-coding splice variants. Colored squares mark the coding gene regions for NMU25, NMS, NURP and NSRP. Aminoacid sequence fragment of individual ppNMU variants encoding NMU25 or NURP were also visualized **(B)**.

In humans both *ppNMU* and *ppNMS* are composed of 10 exons and 9 introns (**Figure 1A**). According to the ENSEMBL genome database, the primary mRNA for *ppNMU* undergoes alternative splicing, leading to the six alternative splice variants of mature *ppNMU* mRNA, including: *ppNMU* var.*1* - 816 nt, (174 aa - primary ppNMU form encoding mature NMU25 peptide an NURP - **Figure 1B**), *ppNMU* var.*2* - 693 nt, (147 aa, encoding mature NMU25 and NURP fragment), *ppNMU* var.*3* - 658 nt, (149 aa, encoding 21 amino acids of NMU and NURP fragment), *ppNMU* var.*4* - 283 nt (transcript variant does not contain translated open reading frame), *ppNMU* var.*5* - 756 nt (158 aa - encoding mature NMU25 peptide an NURP), *ppNMU* var.*6* - 616 nt. (transcript variant does not contain translated open reading frame). It should be emphasized that *ppNMU* and *ppNMS* genes are located on different chromosomes (*ppNMU* on 4q12 and *ppNMS* on 2q11.2) (ENSEMBL) (**Figure 1C**).

In the rat, the *ppNmu* gene comprises 10 exons positioned on chromosome 14. There are discrepancies between the number of alternative splice variants reported in the ENSEMBL and NCBI databases. According to NCBI database there are four alternative splice variants of rat *ppNmu* described as: “neuromedin U (Nmu), mRNA” (708 nt, 174 aa- primary rat ppNmu form), “transcript variant X1” (1107 nt, non-protein coding), “transcript variant X2 “ (966 nt, 164 aa),”transcript variant X3” (1077 nt, non-protein coding). In ENSEMBL database only one splice variant is presented that correspond to “neuromedin U (Nmu), mRNA” - primary rat *ppNmu* form (NCBI). As in humans, the *ppNms* gene contains 10 exons and has a single transcript whose length is 496 nt and 152 aa. This gene is localized on chromosome 9.

In the mouse, the *ppNmu* gene also contains 10 exons, but is located on chromosome 5. This gene has a single splice variant whose length is 828 nt and 174 aa. In the mouse, the NMS gene is also composed of 10 exons. As reported in the NCBI database this gene has 2 alternative splice variants: “transcript variant 1” - 1019 nt, 153 aa; “transcript variant 2” - 995 nt and 145 aa. These two transcript variants are also found in the ENSEMBL database.

The information given above about the structure of genes encoding *ppNMU* and *ppNMS* and their transcripts indicates significant species differences in *ppNMU* and* ppNMS* gene structure. This is reflected in the large number of NMU isoforms described in various animal species. In contrast to NMU, the structure of the NMS gene and the number of its transcripts are less variable across species.

### ppNMU and ppNMS Derived Peptides

**Figure 2:**
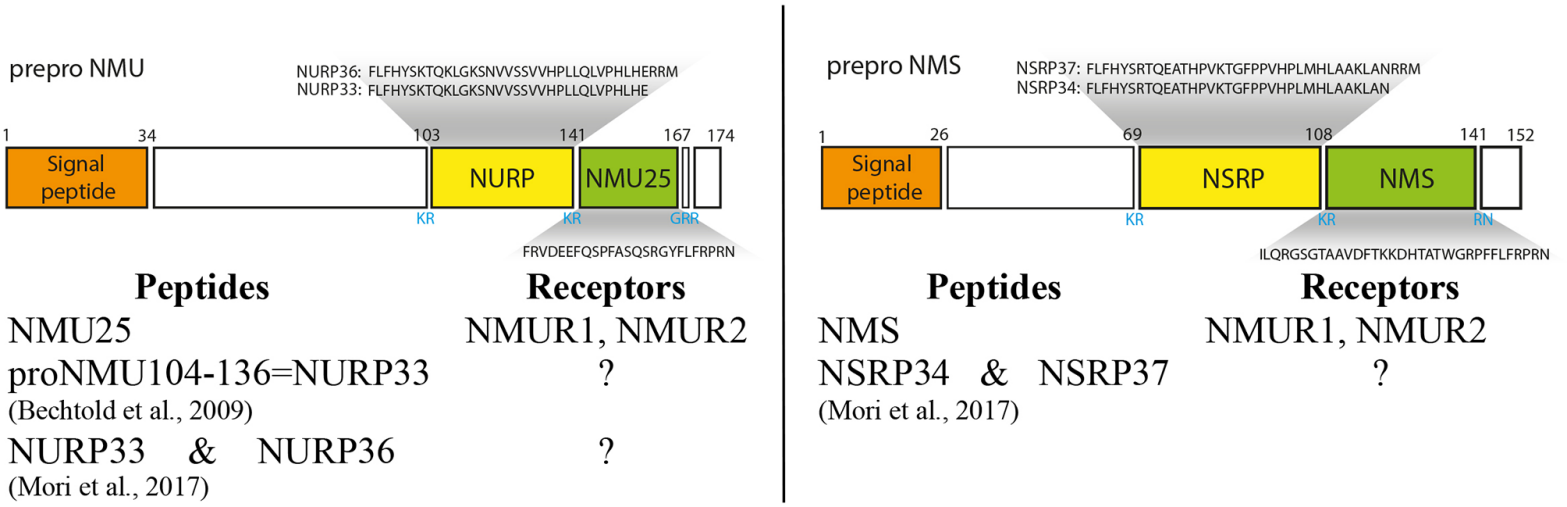
Schematic structure of preproNMU and preproNMS in humans and preproNMU and preproNMS derived peptides. Data from Protein Knowledgebase (UniProtKB) P48645 and Q5H8A3, respectively. Schematic structure of preproNMU is modified from Austin et al. (14). Numbers refer to residues and cleavage sites are given in blue. Authors of the first description of NURP and/or NSRP are shown (5, 8).

In human ppNMU is cleaved into the following 3 chains: neuromedin U25 (NMU25), neuromedin U precursor-related peptide 36 (NURP36) or neuromedin U precursor-related peptide 33 (NURP33) (**Figure 2**) (ENSEMBL). On the other hand ppNMS is cleaved into: neuromedin S33 (NMS33), neuromedin S precursor related peptide 37(NSRP37) and neuromedin S precursor related peptide 34 (NSRP34). In the rat, the NMU is composed of 23 amino acid residues and the NMS of 36. In the mouse, the corresponding numbers are 20 and 36 amino acid residues.

In the mammalian NMUs a common C-terminal sequence – Phe-Leu-Phe-Arg-Pro-Arg-Asn-NH2 –contains the active site of the neuropeptide, which is formed by the amino acid residues between positions 2 and 8 (15, 16). Available data indicate that in most species studied, the five amino acids at the C-terminus of the NMUs are totally conserved, suggesting that this region is of major importance for biological activity (2, 10, 12, 17–19). NMU and NMS are structurally related neuropeptides. They share the same amidated C-terminal heptapeptide and bind to the same receptors NMUR1 and NMUR2 (3, 4).

Other peptides derived from ppNMU and ppNMS, namely NURP36, NURP33, NSRP37 and NSRP34 exert some biological effects, however, they do not act through either NMUR1 or NMUR2 (7). Their biological effects will be presented in more detail in subsequent sections.

## NMU and NMS Receptors

The first report of the presence of a highly specific 125I-NMU binding site in cell membranes obtained from rat uterus suggested that it was a single class of binding site with a Kd of 0.35 nM (20). Soon, two NMU receptors were identified using modern molecular biology techniques (21). Through the efforts of many research groups, the two genes were quickly characterized and their expression in different species and different tissues and organs was identified (22–29). After several years, these receptors were named NMUR1 and NMUR2. NMUR1 expression is mainly observed in peripheral tissues and organs, while the highest expression of NMUR2 occurs in the central nervous system (CNS) [for review see (2, 10, 13)]. Both receptors are typical GPCRs, with characteristic seven transmembrane domains.

In humans NMUR1 gene consists of 3 exons and 2 introns, the size of encoded mRNA is 3274 bp and the receptor is composed of 426 aa residues. Current data from NCBI contain information on the presence of a total of six alternative splice variants of this gene (neuromedin U receptor 1 and transcript variants X1-X5 - encoding 403, 376, 370, 333, 313 aa length, respectively) while the Ensembl database contains information on one isoform. NMUR2 gene, on the other hand, consists of 4 exons and 3 introns. The size of its mRNA is 2067 bp and the receptor is composed of 415 aa residues. In the NCBI there are only 2 splice variants for this gene (neuromedin U receptor 2 and transcript variant X1 encoding 321 aa). NMUR1 gene is located on human chromosome 2 - position q37.1 and NMUR2 gene on chromosome 5 - position q33.1 (10). There are two splice variants of the NMUR1 gene and single transcript of the NMUR2 gene in the rat and mouse.

The high number of NMUR1 and NMUR2 alternative splice variants may suggest that they may be involved in some, as yet unexplained, way in exerting biological effects by NMU, or NMS. The organ specificity regarding the expression of different receptor isoforms is also not elucidated.

Regarding the isoforms of the aforementioned receptors, very interesting are the results of a study on the expression of NMUR1 and NMUR2 in ovarian cancer in women (30). The group identified a truncated NMUR2 in this cancer, which they named NMUR2S. This receptor is a protein with six trans-membrane domains, and it does not bind NMU, but it does modify NMU activity.

NMU shows very high affinity for both receptors (NMUR1 and NMUR2). The biological effects induced by the endogenous ligand (NMU) are being observed already at nanomolar (nM) concentrations [for review see (2, 10, 13)]. NMS also shows high affinity for both receptors. The binding of labeled NMS to human NMUR1 is similar for NMU and NMS. However, for NMUR2, the binding of NMS is significantly higher than that of NMU (3).

In the current literature, several publications indicate the possibility of another receptor(s), in addition to NMUR1 and NMUR2, through which NMU can exert biological effects (7, 8, 12). These are mainly based on studies on the role of NMU in the immune system. Such an example is the arthritis model (31), or the occurrence of NMU-mediated inflammation in NMUR1 and NMUR2 gene knockout mice (32).

Another so far unexplained phenomenon is the mechanism of NURP/NSRP action. Due to their structure, NURP and NSRP cannot activate either NMUR1 or NMUR2, but they exert multiple effects (including stimulation of prolactin release, thermoregulation) (7). These data suggest the presence in cells of receptors other than NMUR1/NMUR2, or alternative signaling pathways through which NURP/NSRP act.

## NMU and NMS Evoked Intracellular Signaling

As mentioned above, NMUR1 and NMUR2 receptors belong to a broad group of GPCRs. Upon ligand binding, the receptors change their conformation and transduce the signal into the cell by acting on heterotrimeric G-proteins, which consist of α-, β-, and γ-subunits. The major determinant of GPCR specificity is Gα, in which four classes of proteins are distinguished, namely Gαs, Gαq, Gα12/13, and Gαi/o (33, 34).

In the case of NMUR1 and NMUR2 receptors, it was demonstrated early on that their stimulation with endogenous ligands leads to increased intracellular Ca^++^ concentration, inositol phosphate generation, inhibition of forskolin-stimulated cAMP generation and ERK (MAPK/ERK pathway) activation (2, 18, 22–29, 35, 36). These data were obtained mainly from experiments on HEK-293, COS-7 and CHO cell lines transfected with NMUR1 or NMUR2 genes. Multiple experiments using inhibitors of specific signaling pathways were performed on these models, which allowed specifying the intracellular changes induced by ligand binding to receptors. However, the results obtained often depend on the experimental conditions applied.

In HEK-293 cells transfected with the NMUR1 gene, receptor activation induced by the addition of NMU does not alter cAMP levels, suggesting that in the cells tested, the Gαi and Gαs subunits are not involved in NMUR1 signaling (23). On the other hand, in CHO cells transfected with the NMUR2 gene, NMU partially inhibits forskolin-induced cAMP synthesis. However, subsequent studies in NMUR1 or NMUR2 gene-transfected HEK-293 cells provided further evidence for the inhibitory effect of NMU-stimulated receptors on forskolin-induced cAMP synthesis (18, 36). It should be noted that this inhibition is stronger when NMUR2 is stimulated.

Along with the identification of NMS as an endogenous ligand of NMUR1 and NMUR2, Mori’s group showed that compared to NMU, NMUR2 has a higher affinity for NMS (3). Further studies using Gαs chimeras showed that in recombinant 293T cells NMUR1 stimulation is mainly transmitted through Gαq, whereas NMUR2 stimulation is preferentially transmitted *via* Gαi (37).

There is evidence that the signaling pathways induced by NMUR1 and NMUR2 stimulation can be modified by the rate of receptor recirculation in the cells tested. Studies performed on HEK-293 cells transfected with the human NMUR2 gene showed that NMU and NMS exhibit very similar acute stimulatory effects on intracellular Ca^++^ concentration (38). Resensitization of this receptor involves its internalization and subsequent acidification in endosomes. Comparing with NMS, the rate of resensitization of NMUR2 is shorter after exposure to NMU. Also, the acute activation of ERK by NMU and NMS is similar; however, it persists longer after NMS stimulation.

Another mechanism for NMU-induced modification of signaling pathways was described by Lin et al. (30). It is related to the NMUR2S receptor they described in ovarian cancer in women. This truncated receptor does not bind NMU but down regulates its action by forming heterodimers with NMUR1 or NMUR2.

The vast majority of studies of signal transduction and intracellular signaling pathways induced by NMU, or NMS, are based on studies of cell lines, mainly transfected with NMUR1 and NMUR2 receptors. However, there are now a growing number of publications about the occurrence of the same/similar phenomena in unaltered human, rat, or mouse cells and tissues (for example various smooth muscle preparations, vascular rings or isolated cells and organs) (19, 39, 40).

## Organ and Tissue Distribution of NMU, NMS, and Their Receptors

Soon after the identification of NMU, its distribution was described in various species, including human, rat, and mouse. The first studies were based on the determination of peptide concentrations in tissue extracts by RIA (41–43). This was later joined by descriptions of NMU distribution based on immunohistochemical methods, *in situ* hybridization (ISH) and Northern blot analysis, or finally QPCR (22, 29). These results concordantly described the highest concentrations of NMU protein and its mRNA in the gastrointestinal tract, spinal cord and central nervous system (CNS) (19).

The distribution of NMUR1 and NMUR2 receptors, which were described in 2000, was already based almost exclusively on studies of mRNA expression levels by QPCR. These have been described in human (24, 25, 27–29) and rat (22, 23, 44). The results obtained consistently report the presence of NMUR1 in the periphery and NMUR2 in the CNS. It should be noted that this localization parallels the distribution of NMU.

Compared to NMU, NMS mRNA expression has a more restricted distribution. In the rat, NMS mRNA expression is mainly observed in the brain, especially in the hypothalamus (here immunoreactive NMS protein is also present), also in the testes and spleen (3, 45).

**Figure 3:**
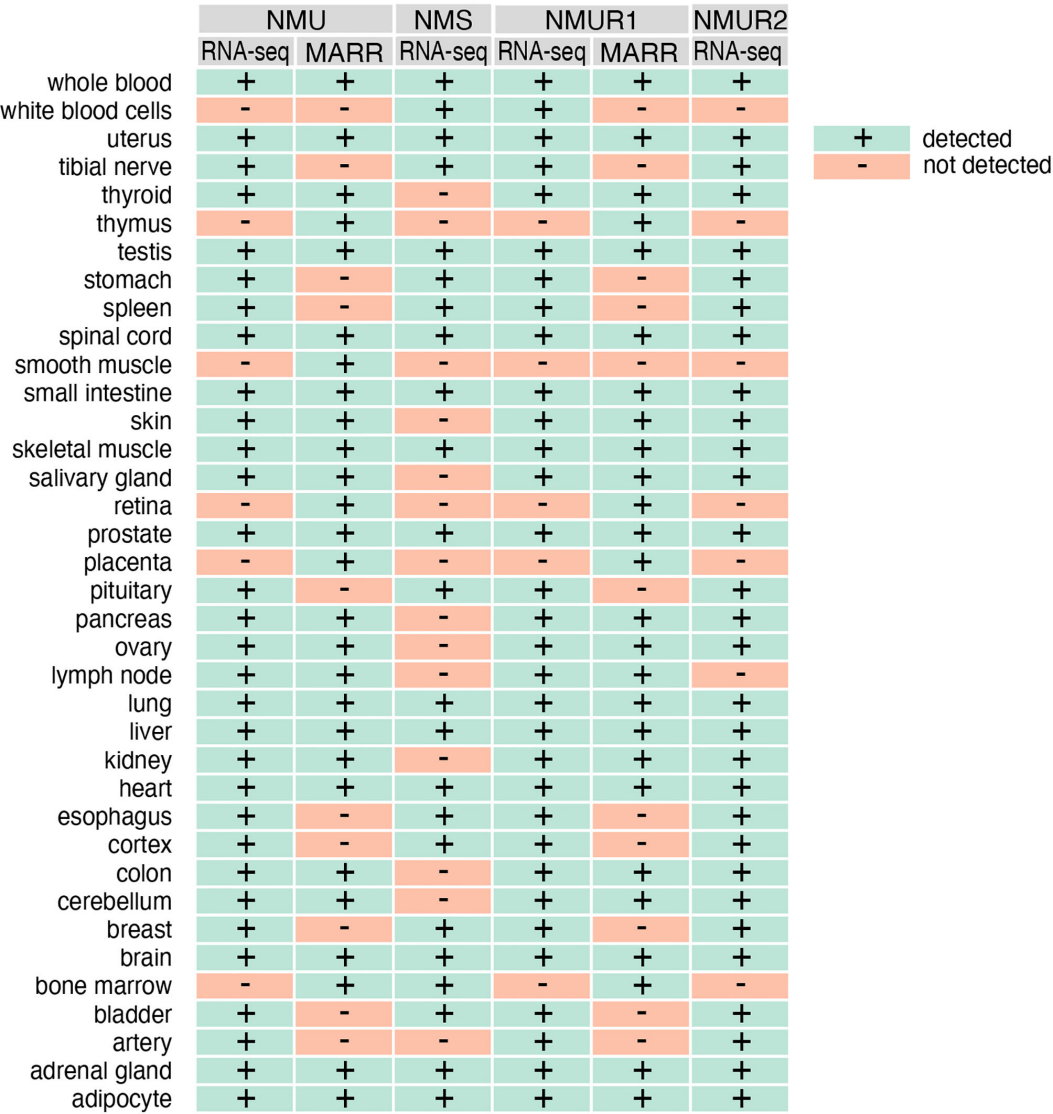
The expression of NMU, NMS, NMUR1 and NMUR2 genes in normal human tissues as assessed by RNAseq and microarray (MARR) methods. Data obtained from gene card analyses. GeneCards identifiers: NMU - GC04M055595; NMS - GC02P100453, NMUR1 - GC02M231572; NMUR2 - GC05M152391.

The introduction of new research methods such as microarrays or NGS (next-generation sequencing) largely confirms the previously reported distribution of NMU, NMS and their receptors NMUR1 and NMUR2 in various human tissues and organs. In this aspect, we performed gene expression analysis of NMU, NMS, NMUR1 and NMUR2 from the GeneCards database. As shown in **Figure 3**, the expression of these genes overlaps significantly with earlier data.

This analysis also shows that the RNA-seq method identifies more organs with NMU gene expression when compared with microarray analyses. But the data obtained from RNA-seq indicate a wider expression of the NMS gene in humans than previously described. Expression of this gene was identified in whole blood, leukocytes, uterus, tibial nerve, testes, stomach, spleen, spinal cord, small intestine, skeletal muscle, prostate, pituitary gland, lung, liver, heart, esophagus, cortex, breast, brain, bone marrow, bladder, adrenal gland, adipocyte, among others. As is well known, NMUR2 is described as a central receptor. However, the results of RNA-seq analyses presented in the mentioned database indicate a much wider mRNA expression of this gene in humans. Of the 37 organs and tissues examined, expression was not observed in leukocytes, spleen, thymus, smooth muscle, retina, placenta, lymph node, and bone marrow only.

**Figure 4:**
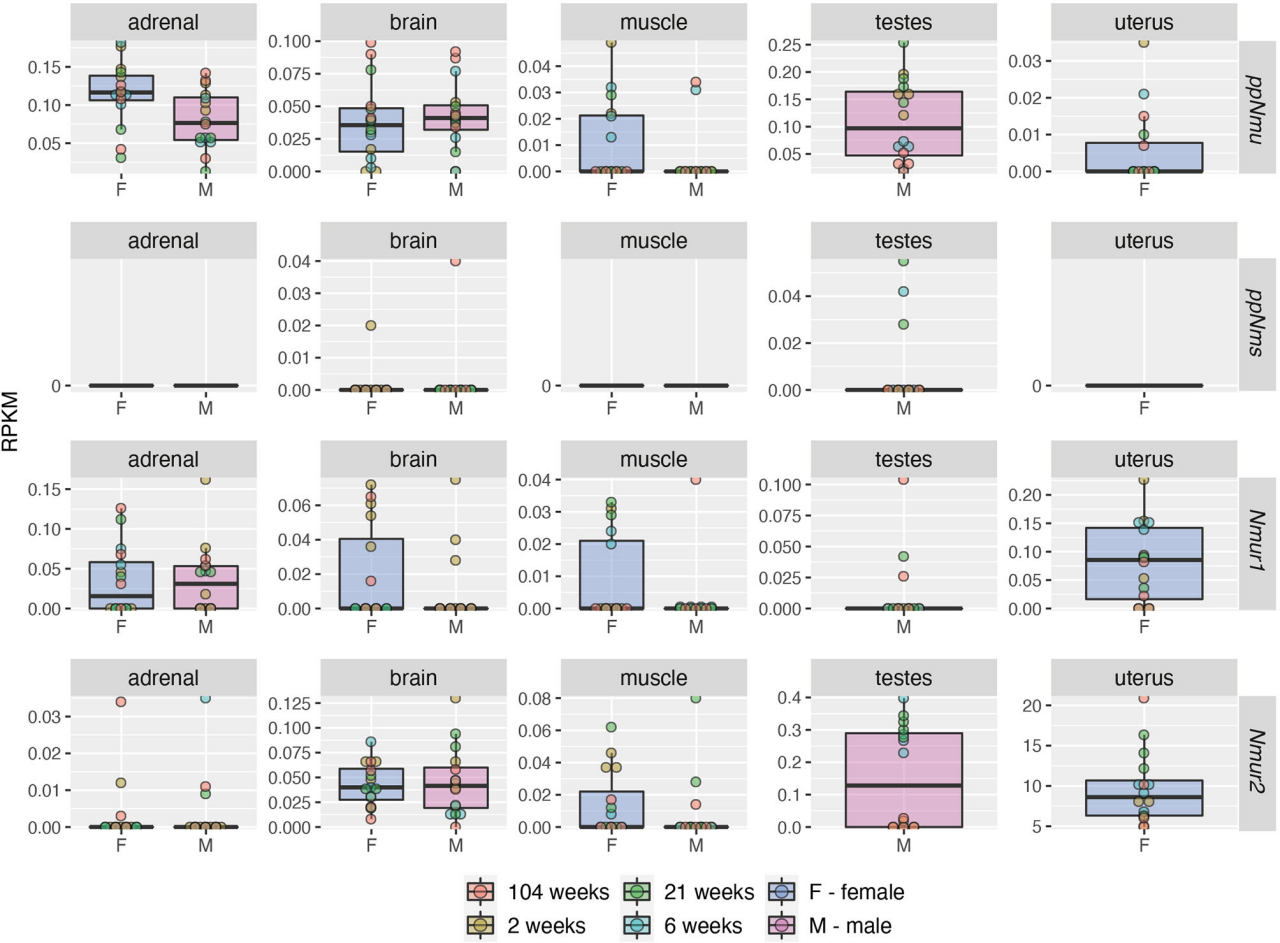
Expression levels (RPKM—reads per kilobase million) of NMU, NMS, NMUR1 and NMUR2 genes in adrenal, brain, muscle, testes and uterus of male and female rats of the Fischer strain in four developmental stages: juvenile (2 weeks), adolescence (6 weeks), adult (21 weeks) and aged (104 weeks) as demonstrated by RNA-seq. Color of the circle corresponds to the appropriate development stage. The analysis of data published by Yu et al. (46). Data was obtained from Gene Expression Omnibus database, accession number: GSE53960 (available at https://www.ncbi.nlm.nih.gov/geo/query/acc.cgi?acc=GSE53960) (accessed date: 4 April 2021).

Much less data based on the RNA-seq method is available for the rat. In this aspect, the data of Yu et al. are worth mentioning (46). In both sexes of Fischer 344 strain rats, this group developed a “transcriptomic BodyMap” covering 11 organs of juvenile, adolescent, adult and aged animals. From the data available in the Gene Expression Omnibus we analyzed the expression of NMU, NMS, NMUR1 and NMUR2 genes in adrenal, brain, muscle, testes and uterus (**Figure 4**). The presented data show that in the adrenal glands of the studied rats there is an expression of NMU and NMUR1 genes, while this expression is absent in relation to NMS and NMUR2. These authors observed a high expression of NMU, NMUR1 and NMUR2 genes in the brain of the studied rats, while a relatively low expression of the NMS gene. In muscles, a very low expression of the NMS gene is noteworthy. In the testes of the studied rats, the authors showed expression of all the studied genes, while in the uterus they did not show NMS transcripts.

Data resulting from RNA-seq analyses of human and rat, as well as data obtained by QPCR, indicate organ-specific expression of the identified transcripts. This expression is also sex and age dependent.

## Functions of ppNMU and ppNMS Derived Peptides

### Central Energy Homeostasis

The identification of the NMU receptors was linked to a significant discovery of the peptide’s action, which to this day remains a subject of interest to many research centers. It appeared that administered intracerebroventrical (icv) NMU, in different species reduces food intake and body weight, a similar phenomenon occurs when NMU is injected into the paraventricular nucleus (PVN). This effect is observed in rats (25, 26, 47–51). In mice, on the other hand, silencing of the NMU gene increases food intake and leads to obesity (52), while the opposite effect is observed in animals with overexpression of this gene (53). The effects of icv administered NMU do not occur in NMUR2 gene knockout mice (54).

In humans, two NMU variants have been identified that suggest the altered structure of the peptide may lead to metabolic changes manifested by the onset of obesity (55). One mutation occurs in the ppNMU signal peptide (NMUAla19Glu), while the other occurs within the active peptide (NMUArg165Trp).

In the search for a mechanism of action of NMU on energy homeostasis of the organism, the earliest interest was focused on corticotropin releasing hormone (CRH). It has been known for years that CRH, whose expression is high in PVN, inhibits food intake in the rat (56). Subsequent studies demonstrated direct NMU effects on CRH and arginine vasopressin (AVP) release by rat hypothalamic explants *in vitro* (50). On the other hand, results of whole cell patch-clamp recordings revealed that NMU directly depolarizes the subpopulation of PVN parvocellular, but not magnocellular neurons *via* hyperpolarization-activated inward current enhancement (57). Moreover, NMU-sensitive neurons expressed NMUR2 mRNA (58). Direct effects of both neuromedins on PVN was also suggested by *in vitro* electrophysiological studies which showed that NMU and NMS increased the neuronal firing rates in both arcuate (ARC) and PVN nuclei slices (59). Likewise, studies in wild-type mice and in CRH gene-silenced mice suggest that NMU-induced food intake is mediated through CRH (51, 60).

Leptin is another peptide that has received considerable attention in the aspect of inhibitory effects of NMU on food intake and associated weight loss. Leptin, acting at the hypothalamic level, exerts anorexigenic effects, also modifies the function of the neuroendocrine or immune systems (61–63). Furthermore, it should be noted that leptin stimulates NMU release from rat hypothalamic explants (50). This observation suggests that “NMU acts downstream of leptin” (64). However, icv administration of leptin to mice with a silenced NMU gene reduces their body weight (52). This latter observation suggests that the anorexigenic effect of NMU, at least in mice, is not related to the action of leptin.

As described by Mori et al. (3), the highest level of NMS mRNA expression occurs in the hypothalamus, and as shown by studies using ISH, this neuropeptide has a specific localization in the suprachiasmatic nuclei. It is interesting to note that in the rat hypothalamus expression of NMS gene is nearly 3-fold higher than that of NMU gene (65). The group that identified NMS described anorexigenic effects of NMS in mice the same year (66). They found that NMU decreased food intake when administered *via* the icv route, an effect accompanied by increased levels of POMC mRNA expression in the ARC and CRH mRNA expression in the PVN. Anorexigenic effects of NMS were also observed in Japanese quail (67). As suggested by studies in mice, the anorexigenic effects of icv administered NMU and NMS are mediated primarily through NMUR2 (8, 68). The inhibitory effect of NMS on food intake in mice is accompanied by a decrease in gastroduodenal motility (69).

Detailed comparisons of NMU and NMS effects on food intake in rodents indicate that their anorexigenic effects are very similar (59). In their study, the authors evaluated the effects of the tested peptides administered icv on CRH mRNA expression level in NPV and POMC mRNA in ARC. In addition, both peptides stimulated the secretion of α-melanocyte-stimulating hormone in the ARC tissue culture.

NURP and NSRP also exert effects on energy homeostasis and food intake in rats and mice. In mice, icv administration of NURP33 leads to a transient (∼4 h) increase in food intake followed by a decrease (8). This peptide also increased the metabolic rate of the examined animals. Further studies have shown that icv administration of NURP33, like NMU and NURP36, leads to an increase in locomotor activity and energy expenditure in rats, but NURP33 does not reduce food intake (6). As is known, both NURP33 and NURP36 do not activate NMU receptors, hence the authors suggest that both peptides “might exert NMU-like sympathetic nerve action in the brain”. Subsequent studies have shown that NSRP administration has no effect on food intake, although examined rats’ body temperature and locomotor activity increased under these conditions (7). The authors also point out that compared to NURP, the NSRP-induced effects were much weaker. The mechanism of action of both peptides (NURP and NSRP) is not elucidated, their biological effects may occur through as yet unidentified receptors or through their interference with non-identified intracellular signaling pathways.

### Thermoregulation

As defined by Szekely et al. (70) “thermoregulation means a fast energetic adjustment to secure a relative thermal stability of the body”. The aforementioned authors emphasize that in the CNS, anabolic (orexigenic) neuropeptides increase food intake and have a general tendency to induce hypothermia, whereas catabolic (anorexigenic) neuropeptides reduce food intake and enhance energy expenditure with a tendency to induce hyperthermia. In this aspect, it should be noted that early on in the study of the physiological role of NMUs, it was repeatedly described in both the rat and mouse that NMU-induced reductions in food intake are accompanied by increases in body temperature. For example, in the rat, icv administration of NMU exerts such effects (47, 60, 71). Subsequent studies of the mechanism of the hyperthermic effect of NMU in the rat have identified mediators of this effect. Upon icv administration, NMU increased colon temperature, and this effect was blocked by a variety of inhibitors (72). Analysis of the action of these inhibitors suggests that the hyperthermic effect of the neuromedin studied is mediated by CRHR1 and CRHR2, dopamine and muscarinic cholinergic receptors, and finally prostaglandins.

Although an earlier publication did not reveal this, NMS is also involved in the regulation of thermogenesis (73). However, subsequent detailed studies have shown that in the rat, both NMU and NMS administered icv increase the back surface temperature (74). Furthermore, based on similar measurements in mice with silenced NMU and/or NMS genes exposed to low temperature, the authors suggest that not NMU but endogenous NMS plays a dominant role in regulating thermogenesis. They also found that the mediators of these phenomena are factors previously described by Telegdy and Adamik (72).

Recent studies have shown that NURP and NSRP are also involved in the regulation of thermogenesis (6, 7). These authors demonstrated that NURP and NSRP act in different regions of the rat brain than NMU and NMS. Furthermore, both peptides (NURP and NSRP) administered icv increased the back surface temperature, with the effects induced by NSPR being weaker.

### Control of Circadian Rhythms

The circadian oscillator system in animals that forms the autonomic biological clock is formed by the hypothalamic suprachiasmatic nucleus (SCN) and clock genes present in all cells (75–77). The SCN times these peripheral clock genes. It is important to note that even in culture, SCN neurons retain the properties of a biological oscillator (78).

The first observations on the involvement of the NMU - NMU receptors system in the regulation of SCN function were carried out by Nakahara et al. (79). These authors demonstrated the expression of NMU, NMUR1, and NMUR2 mRNAs in the rat SCN, and the expression levels of NMU and NMUR1 mRNAs fluctuated with circadian rhythm. The results of these studies suggested that NMU, through an autocrine or paracrine pathway, may regulate SCN function. Also using the ISH method, it was shown in C3H strain mice that the level of NMU mRNA expression in the SCN varies with circadian rhythm (80). In terms of circadian rhythm control, the observations by the team of Mori et al. (3) related to NMS identification were pioneering. This team demonstrated high levels of NMS mRNA expression in the SCN of the rat, with the level of this expression altering in circadian rhythm, but not changing in darkness.

Many interesting insights into the role of NMUs and NMS in regulating circadian rhythms were provided by the research of the team of Lee et al. (81). In a study on C57BL/6J mice, using genetic engineering methods and genetic crossbreedings, this team showed that NMS neurons “are capable of dictating behavioral circadian period”. However, silencing the Bmal1 gene in SCN neurons, which is a major transcription factor that regulates the mammalian biological clock, abolishes behavioral circadian rhythms. Interestingly, silencing of NMU or NMS genes does not result in loss of circadian rhythms. It remains an open question whether similar phenomena occur in rats.

### Stress Response

Independently of mode of administration (icv, iPVN, or sc) NMU activates CRH containing neurons and stimulates CRH secretion, which in turn triggers pituitary ACTH and adrenal corticosterone/cortisol secretion. In this regard it is not astonishing that NMU and NMS are involved in central and peripheral control of the stress response. These studies were initiated by Hanada et al. (60) who observed stress-related behavior (gross locomotor activity, face washing and grooming) in icv NMU administered rats. This response was attenuated by pretreatment with alpha-helical CRH (antagonist of CRH) or anti-CRH IgG. Furthermore, NMU-induced increases in oxygen consumption and body temperature were attenuated in CRH KO mice. Subsequent studies demonstrated that blood corticosterone levels were significantly increased after 10 min of immobilization stress in wild-type mice, but not in NMU KO mice (79). Excessive grooming induced by icv NMU administration were abolished in the NMUR2 KO mice, an observation suggesting that NMUR2 plays a decisive role in stress/anxiety induction (54).

It was only after a long gap that another publication was published presenting results of studies on the role of NMU8 in stress (82). The study was conducted in male C57BL/6J mice that were administered the neuropeptide icv. The obtained results indicate that in mice, NMU8 regulates the response to forced swim stress, whereby the effect induced by NMU8 was dependent on previous exposure to stress.

### Endocrine System

The role of NMU and NMS in regulating endocrine function has been described by us previously (11, 83). These issues have also been widely discussed in other review publications (2, 4, 9, 10, 12, 13). Therefore, in the current review, we focus on recent reports related to the role of pNMU- and pNMS-derived peptides in endocrine function.

As can be seen from the data presented above, at the hypothalamic level, both NMU and NMS exert significant effects on CRH synthesis and release, and this effect is mediated by NMUR2. Therefore, in this section of the review, we omit the hypothalamic data and focus on the pituitary and peripheral endocrine glands. However, it should be noted that NMU directly stimulates CRH release from rat hypothalamic explants (50).

#### Pituitary Gland

The earliest studies demonstrated high levels of NMU-like immunoreactivity in the rat pituitary gland, and high expression of NMU and NMUR2 genes is also observed in this gland (65, 84). Electron microscopy studies revealed NMU-like immunoreactivity in some thyrotropes and most corticotropes of the rat pituitary gland. NMU is colocalized with galanin and ACTH in the same secretory granules (85) [for review see (86)]. NMU mRNA is expressed in pituitary gland of WT mice, but in contrast, NMU mRNA could not be detected in NMU KO mice (87).

Along with the identification of NMS, the description of the distribution of this neuromedin includes data indicating expression of the NMS gene in the rat pituitary gland (3). However, in this gland, the expression level of the NMS gene was notably lower than that of NMU gene (65).

Conflicting data were reported on expression of NMUR1 and NMUR2 in pituitary gland. By means of QPCR low expression of both receptors in human pituitary gland was reported by Raddatz et al. (27). These data were confirmed by other groups (28, 88). The earliest studies did not reveal NMUR1 gene expression in the rat pituitary gland while that of NMUR2 was very low (22, 88). On the other hand, in rat adenohypophysis expression of NMUR1 gene, but not of NMUR2 was observed by our group (65). Expression of both NMUR1 and NMUR2 genes was observed in mouse pituitary gland of both WT and NMU KO mice (87)

All publications describing the central action of NMU on the hypothalamo-pituitary-adrenal (HPA) axis, both in the rat and mouse, indicate a stimulatory effect of neuromedin on CRH secretion in the hypothalamus, leading to an increase in ACTH and consequently corticosteroid secretion. In the rat, a similar effect is exerted by NMS, which acts through the CRHR1 receptor (73).

Only a few reports have addressed the direct effects of NMU on pituitary hormone secretion. Among others, Fukue et al. (87) demonstrated that NMU inhibits LH secretion from rat anterior pituitary primary cell cultures of female Sprague-Dawley rats. In the same study, FSH secretion showed a decreasing trend.

Also, NURP is involved in the regulation of pituitary function (5, 89). Using the RIA method, this group demonstrated very high levels of immunoreactive NURP in the rat pituitary gland, while much lower levels of this substance are found in the small intestine and brain. When administered icv, NURP increased blood levels of prolactin without altering the levels of other hormones of the anterior lobe of the pituitary gland. However, NURP had no effect on prolactin release from dispersed anterior pituitary cells. These observations suggest that in the rat, NURP stimulates prolactin secretion *via* an indirect pathway, probably *via* dopamine. In contrast to NURP, icv administered NMU significantly decreased prolactin secretion in rats with different types of hyperprolactinemia. These observations suggest that the reciprocal relationship between NMU and NURP may be involved in the physiological regulation of prolactin secretion.

#### Thyroid Gland

Only a few publications deal with the presence and role of the NMU/NMS system and their receptors in the thyroid. The immunoreactive NMU content in the rat thyroid is very low; thyroxine administration does not alter it, but administration of antithyroid agents decreases it (90). NMU-immunopositive substances are found in a small group (∼5%) of parafollicular C-cells, while no staining was observed in nerve fibers. NMU and NMUR1 mRNA expression is detected in the rat thyroid, whereas NMUR2 mRNA expression is very low (65, 88). In the rat thyroid gland, expression of NMU gene is almost 1000-fold higher when compared with the expression of NMS gene. The functional relevance of NMU and NMS in the thyroid gland remains completely unknown. To our knowledge, only two publications have examined the effects of NMU on thyroid, or thyrotropin (TSH) levels. In the first of these Gartlon et al. (88) reported that in the rat icv NMU administration did not affect plasma thyrotropin (TSH) levels. In the second, Helfer et al. (91) have demonstrated that NMU increases the expression level of type II deiodinase, an enzyme responsible for converting inactive thyroid hormone into its active form, in the rat thyroid gland.

#### Adrenal Gland

Following the identification of NMU, it was shown very early on that this neuromedin at the level of the hypothalamus stimulates CRH secretion, which in turn leads to stimulation of ACTH and subsequently corticosteroid secretion. Recognition of the role of NMU/NMS in stress has greatly increased interest in this peptide, resulting in a number of publications explaining various aspects of their action at the level of the adrenal gland as an anatomical unit as well as at the cellular level.

Potent stimulating effects of exogenous NMU on adrenocortical steroid secretion in the rat have been described as early asin 1993. A single sc injection of NMU resulted in a transient increase in ACTH blood concentration (between 3 and 12 h) and a sustained (24h) elevation of plasma corticosterone concentration (92, 93). These data demonstrated stimulating effect of neuropeptide on adrenal cortex, possibly partially due to the direct effect of NMU on the gland.

In the adrenal glands, both human and rat, expression of the NMU gene is found, whereas by QPCR no or very low expression of the NMS gene is demonstrated. In the adrenal glands, expression of the NMUR2 gene mainly occurs, while expression of the NMUR1 gene is negligible (22, 23, 27, 28, 65). NMUR1 mRNA was detected in all adrenocortical zones and in medulla of the gland (94). Moreover, the presence of NMUR1 mRNA in isolated zona glomerulosa and fasciculata/reticularis cells rules out the possibility that the expression was due to the presence in the specimens assayed of the non-parenchymal components of the gland. It should be noted that in the human adrenal gland, the highest density of 125I-NMU25 binding sites is observed in the zona glomerulosa and reticularis, and slightly lower in the zona fasciculata and medulla (19). Expression of NMUR1 as mRNA and protein was demonstrated in adrenal gland of intact rat, in enucleation-induced regenerating gland, in hemiadrenalectomized animals (compensatory adrenal growth) as well as in ACTH-stimulated one (95–97). Furthermore, NMU had no effect on basal and ACTH-stimulated corticosterone secretion by freshly isolated or cultured inner zone adrenocortical cells, nor did it change their cytosolic Ca^++^ concentration (93, 94). However, this neuropeptide stimulated corticosterone output by adrenal slices, but not by fragments of adrenocortical autotransplants lacking medullary chromaffin cells (98).

The results of subsequent studies on the mechanism of action of NMU on steroidogenesis in the rat adrenal gland led to the hypothesis that stimulation of corticosteroid secretion by NMU in the rat adrenal gland is mediated through the medulla of the gland (98). These studies suggested that NMU stimulates adrenaline secretion from medullary chromaffin cells, a hormone that can modulate corticosteroid secretion [for review see (99–101)]. In this regard, it should be noted that it has been repeatedly reported that centrally administered NMU stimulates adrenal medullary secretion of adrenaline [for example (102)].

Neuromedin U also directly regulates adrenocortical growth; it stimulates the proliferative activity of immature rat inner adrenocortical cells in primary culture (94). NMU is also involved in the regulation of adrenal growth *in vivo*, however, its effect is dependent on the type of growth (103). In enucleation-induced adrenal regeneration NMU notably enhanced proliferative activity of adrenocortical cells (95). A similar stimulating effect of sc administered NMU on proliferative activity (metaphase index) was seen in ACTH treated rats (97). In contrast, in hemiadrenalectomized rats NMU notably inhibited adrenocortical cell proliferation in both zona glomerulosa and zona fasciculata, as assessed by the metaphase index (96).

NMS, like NMU also stimulates corticosteroid secretion. In rats icv NMS administration resulted in nearly 5-fold increase in plasma corticosterone concentrations and the effect was dependent on neuropeptide dose (73). This effect is consistent with the action of NMU because both neuromedins act through the same receptors.

#### Pancreatic Islets

In recent years, there is an increasing interest in the role of NMU in the regulation of insulin secretion by pancreatic islands. The first report on the expression of NMU/NMS system elements and their receptors in the rat pancreas revealed NMUR1 mRNA expression in the gland (22). In subsequent studies performed on isolated rat pancreatic islets using RT-PCR and Western blotting our group demonstrated expression of NMUR1 but not NMUR2 (104). Furthermore, we have demonstrated that NMU dose-dependently decreased insulin output by isolated pancreatic islets. In subsequent studies on isolated rat pancreatic islets, we also revealed that NMU and NMUR1 genes are expressed at the mRNA level, whereas the expression of NMS and NMUR2 was negligible (65). The inhibitory effect of NMU on insulin secretion in the rat is also observed in an *in situ* pancreatic perfusion model (105). Since NMU stimulates somatostatin secretion in pancreatic islets, we also tried to clarify whether the inhibitory effect of NMU on insulin secretion is mediated through somatostatin. Our results suggest that somatostatin mediates the inhibitory action of NMU on insulin secretion. The inhibitory effect of NMU on glucose stimulated insulin secretion is also observed in human pancreatic islets in both static batch culture and islet perifusion models (106).

Interesting information regarding the effect of NMU on insulin secretion was provided by the study of Zhang et al. (107). These studies were performed on mice and clonal pancreatic β-cell lines (MIN6-K) (108). The authors found expression of NMU and NMUR1 in both pancreatic islets and the MIN6-K8 cell line. Both *in vivo* and *in vitro*, NMU inhibited glucose-induced insulin secretion. In contrast, insulin secretion was elevated in siNMU-transfected MIN6-K8 cells. These results suggest that NMU can act directly on β cells through NMUR1 in an autocrine or paracrine manner.

In regard to the data presented above on the stimulatory effect of NMU on insulin secretion, the results of Kuhre et al. (109) are somewhat surprising. These authors found no changes in blood insulin levels after intravenous injection of NMU into rats. Also, they did not observe NMU-induced effects on insulin secretion by perfused rat pancreas. Furthermore, in both human and rat pancreas, the authors did not detect the expression of either NMUR1 or NMUR2 (QPCR method and ICH).

A new perspective on the insulinostatic role of NMU is presented by the group of Zhang et al. (40, 110). These authors highlight the colocalization of NMU and insulin in pancreatic β-cells. They also showed that NMU induces mitochondrial dysfunction by impairing mitochondrial biogenesis, respiration, and mitochondrial Ca^++^ uptake in β-cell-derived MIN6-K8 cells, causing endoplasmic reticulum stress. In their study, it is noteworthy that 24 h exposure of MIN6-K8 cells to NMU increases their rates of apoptosis. Based on further research, this group suggests that “NMUR1 coupled to pertussis toxin-sensitive Gαi2 and Gαo proteins in β cells reduced intracellular Ca^++^ influx and cAMP level, thereby causing β-cell dysfunction and impairment”.

#### Testis

The expression of the NMU/NMS and their receptors in the testis is the subject of only a few publications. Apart from early reports, which presented the expression of individual elements of this system in various organs, there are practically no reports on their role in the male gonad.

Fuji et al. (22) showed that there is moderate expression of the NMU gene in the rat testis, but negligible expression of the NMUR1 gene. In our study, we observed much higher expression of the NMS gene than the NMU gene at the mRNA level in rat testes, and we also identified the NMS protein (Western blotting) (65). There is moderate expression of NMUR1 gene in rat testes, while the expression level of NMUR2 was negligible. The data on NMS mRNA expression in rat testes is also supported by the study of Miyazato et al. (111),. It should be noted that using a highly specific RIA, Mori et al. (45) showed high levels of NMS protein only in the hypothalamus, while in the testes “it was hardly detected”.

#### Ovary

Similar to the testis, information on the expression and role of the NMU/NMS and their receptor system in the ovary is very scarce. In the rat, Fuji et al. (22) demonstrated a moderate level of NMU mRNA expression in the ovary, whereas NMUR1 mRNA expression was negligible. In our study, we observed a low level of NMU gene expression in rat ovaries, while we did not show NMS gene expression. In the rat ovary, the expression of NMUR1 gene is mainly present, and the expression of NMUR2 gene is very low (65).

NMU gene expression was demonstrated in cell lines IOSE80 and IOSE144, which are derived from human ovarian surface epithelial cells (112). In the search for ovarian cancer-associated antigens, the authors used the expression of the NMU gene in these cells as the basal expression, which was compared with gene expression in various ovarian cancers. They showed that this expression is about 5-fold higher in ovarian cancers.

### Cardiovascular System

Along with the identification of NMU, it has been shown that in the rat this peptide administered intravenously induces an increase in blood pressure (1). This observation has been repeatedly confirmed, with these effects being rather brief and observed after administration of the peptide both centrally and peripherally. Early studies also demonstrated that this peptide is primarily involved in the regional regulation of intestinal blood flow (113).

Following the identification of NMS, research into the role of the NMU/NMS system and their receptors in regulating cardiovascular function has taken on new aspects. The first report indicated that NMS administered icv does not alter heart rate in rats (73).

In the aspect of research on the role of NMU and NMS, the results of human organ studies deserve special mention (19). The authors studied the effects of these peptides on heart, aorta, and coronary artery, mammary and radial artery and saphenous vein, among others. They demonstrated the expression of both NMU and NMS and their receptors NMUR1 and, much lower NMUR2 in the human cardiovascular system. They further demonstrated that the vasoconstrictor effect of NMU is endothelium-independent. A similar effect is produced by NMS. The team demonstrated for the first time the expression of NMU and NMS in the myocardium and its vessels, where NMUR1 receptor expression also occurs. The authors suggest that this system may be involved in the control of hemodynamics.

The above observations indicate a direct action of NMU and NMS in the myocardium and vasculature. In this context, there are relatively numerous reports also of indirect - *via* the sympathetic nervous system - effects of these peptides on cardiovascular function in the rat (114–116).

The direct effect of NMS on rat cardiomyocytes is also evidenced by the direct stimulatory effect of this peptide on L-type Ca^++^ channel currents (117).

A recent report shows that in the H9 cell line (human embryonic stem cell derived cardiomyocytes), in a model simulating cardiac ischemia reperfusion injury NMUR1 is directly involved in protecting the studied cells from damage (118). In the experimental model used, miR-1275 expression is significantly increased. The authors showed that miR-1275 directly targets the 3ʹUTR of NMUR1 and negatively regulates NMUR1 expression. Silence of NMUR1 abolished the protecting effect of the miR-1275 antagomir on myocardial injury.

### Bone

With regard to bone, the first publication on the role of the NMU/NMS and its receptors system was by Sato et al. (119). In mice with a silenced NMU gene, this team demonstrated higher bone mass compared to WT animals. Based on physiological experiments *in vivo* and on cultured osteoblasts, the authors suggest that the changes in bone depend on the action of NMU in the CNS rather than on a direct action of the neuromedin on osteoblasts. The last suggestion comes from studies performed on cultured mouse calvarial osteoblast-like cells exposed to NMU. Under these conditions, alkaline phosphatase activity, mineralization, and expression of osteoblast marker genes were not altered. Moreover, both osteoblasts cultured from NMU-silenced and WT mice proliferated normally in the presence of NMU. Also, osteoclastic differentiation from bone marrow macrophages was unchanged by NMU treatment.

In the aspect of the above results, our group performed a study on the role of the NMU/NMS system and their receptors in regulating functions of cultured rat calvarial osteoblast-like (ROB) cells (120). By using QPCR, high expression of NMU mRNA was found in freshly isolated ROB cells while after 7, 14, and 21 days of culture, expression of the studied gene was very low. In contrast, NMUR2 mRNA expression in freshly isolated ROB cells was negligible and very high in cultured cells. The highest NMUR2 mRNA expression was observed at day 7, and was followed by lower levels at days 14 and 21 of culture. Neither NMS nor NMUR1 mRNA was found in studied cells. Exposure of cultured ROB cells to NMU8 had no effect on expression levels of the genes. During the entire culture period, NMU8 did not affect osteocalcin production, but stimulated proliferative activity of ROB cells at days 14 and 21 of culture.The stimulatory effect of NMU8 on ROB proliferation was not observed on day 7 of culture (proliferative stage of culture).

Of interest are the current findings on the role of NMU in regulating osteoblast differentiation and activity (121). These studies were performed on MC3T3-E1 (osteoblast precursor cell line derived from newborn mouse calvaria, cultured for 9 days) and W-20-17 cells (cell line derived from mouse bone marrow stromal cells, cultured for 17 days). These cells were cultured in the presence of NMU8 and NMU25. In W-20-17 cells NMU expression increased as cells underwent osteoblastic differentiation. Exposure to NMU25 during differentiation led to a reduction in the expression of osteoblast markers, including osteocalcin, whereas this effect was not observed with exposure to NMU8. In cultured MC3T3-E1 cells, both NMU8 and NMU25 increased the expression of osteoblast markers but had no effect on osteocalcin expression levels. In these cells, NMU8 resulted in increased proliferation, whereas NMU25 had no effect on the division of these cells. It appeared that intracellular signaling pathways induced by NMU8 and NMU25 in the tested cells differed. In the W-20-17 lineage, NMU25 stimulates activation-related phosphorylation of multiple effectors, whereas no such effect was exerted by NMU8. In contrast, in MC3T3-E1 cells NMU8 as well as NMU25 evoked very similar effects.

As can be seen from the above data, the effect of NMU on osteoblast function differs significantly, depending on the cells studied, the duration of culture as well as the isoform of NMU used in the study (25 *vs.* 8 aa). The different effects of NMU8 and NMU25 on osteoblasts may also suggest the presence in osteoblasts of another, not yet identified receptor, as we mentioned earlier. This possibility is also suggested by the results of phospho-profiling arrays (121).

## Addendum: NMU in Pathophysiology

In recent years, an increasing number of publications have addressed the role of NMU in pathophysiology, including the role of this neuromedin in immunology and inflammation, as well as in the biology of various cancers. These issues have been presented in several current review papers. Therefore, these topics are not addressed in the present article. Interested readers are encouraged to refer to relevant papers on immunology and inflammation (122–124) and cancers (125).

## Conclusion

The system of NMU/NMS and their receptors occurs practically in the entire body and is associated with the functioning of various organs. Modern techniques of molecular biology have led to the identification of new biologically active peptides of this system, which are NURP and NSRP, whose mechanism of action may be mediated by a yet to be discovered receptor (except NMUR1 and NMUR2). Recent advances in understanding the role of this system are based primarily on genetically modified mice, as well as studies on a variety of cell lines. However, it should be emphasized that the results of these studies often cannot be generalized because they are dependent on experimental conditions. It seems that development of highly specific NMUR1 and NMUR2 antagonists would allow even more precise understanding of the role of NMU and NMS in regulation of physiological processes at the organ and cellular level.

## Author Contributions

MR, LM: conceptualization of the article, manuscript writing, review and editing. All authors contributed to the article and approved the submitted version.

## Funding

This research was supported by: “Sonata Bis Grant” program of the National Science Center No. UMO-2020/38/E/NZ4/00020.

## Conflict of Interest

The authors declare that the research was conducted in the absence of any commercial or financial relationships that could be construed as a potential conflict of interest.

## Abbreviations

aa, amino acid; ARC, arcuate nucleus; CHO, Chinese hamster ovary cells; CNS, central nervous system; COS, fibroblast-like cell lines derived from monkey kidney tissue; CRH, corticotropin releasing hormone; CRHR1, corticotropin-releasing hormone receptor 1; CRHR2, corticotropin-releasing hormone receptor 2; ERK, extracellular signaling-regulated kinase; GPCR, G protein coupled receptor; HEK-293, human embryonic kidney 293 cells; HPA,hypothalamo–pituitary–adrenal; ICH, immunocytochemistry; icv, intracerebroventrical; ISH, *in situ* hybridization; KO, knock out; MAPK, mitogen-activated protein kinase; MARR, microarray; NMS, neuromedin S; NMU, neuromedin U; NMUR1, neuromedin U receptor 1; NMUR2, neuromedin U receptor 2; NMUR2S, neuromedin U receptor 2 splice variant; NSRP, neuromedin S precursor related peptide; nt, nucleotide; NURP, neuromedin U precursor related peptide; PpNMS, preproNMS; PpNMU, preproNMU; PVN, paraventricular nucleus; QPCR, quantitative RT-PCR; RIA, radioimmunoassay; RNAseq, RNA sequencing; sc, subcutaneous; SCN, suprachiasmatic nucleus; SON, supraoptic nucleus; TSH, thyrotropin; WT, wild type.
